# SIRT1 Overexpression Antagonizes Cellular Senescence with Activated ERK/S6k1 Signaling in Human Diploid Fibroblasts

**DOI:** 10.1371/journal.pone.0001710

**Published:** 2008-03-05

**Authors:** Jing Huang, Qini Gan, Limin Han, Jian Li, Hai Zhang, Ying Sun, Zongyu Zhang, Tanjun Tong

**Affiliations:** 1 Peking University Research Center on Aging, Beijing, People's Republic of China; 2 Department of Biochemistry and Molecular Biology, Peking University Health Science Center, Beijing, People's Republic of China; National Institutes of Health, United States of America

## Abstract

Sir2, a NAD-dependent deacetylase, modulates lifespan in yeasts, worms and flies. The SIRT1, mammalian homologue of Sir2, regulates signaling for favoring survival in stress. But whether SIRT1 has the function to influence cell viability and senescence under non-stressed conditions in human diploid fibroblasts is far from unknown. Our data showed that enforced SIRT1 expression promoted cell proliferation and antagonized cellular senescence with the characteristic features of delayed Senescence-Associated β-galactosidase (SA-β-gal) staining, reduced Senescence-Associated Heterochromatic Foci (SAHF) formation and G1 phase arrest, increased cell growth rate and extended cellular lifespan in human fibroblasts, while dominant-negative SIRT1 allele (H363Y) did not significantly affect cell growth and senescence but displayed a bit decreased lifespan.. Western blot results showed that SIRT1 reduced the expression of p16^INK4A^ and promoted phosphorylation of Rb. Our data also exposed that overexpression of SIRT1 was accompanied by enhanced activation of ERK and S6K1 signaling. These effects were mimicked in both WI38 cells and 2BS cells by concentration-dependent resveratrol, a SIRT1 activator. It was noted that treatment of SIRT1-.transfected cells with Rapamycin, a mTOR inhibitor, reduced the phosphorylation of S6K1 and the expression of Id1, implying that SIRT1-induced phosphorylation of S6K1 may be partly for the decreased expression of p16^INK4A^ and promoted phosphorylation of Rb in 2BS. It was also observed that the expression of SIRT1 and phosphorylation of ERK and S6K1 was declined in senescent 2BS. These findings suggested that SIRT1-promoted cell proliferation and antagonized cellular senescence in human diploid fibroblasts may be, in part, via the activation of ERK/ S6K1 signaling.

## Introduction

Cellular senescence, a process of cell aging in which primary cells in culture lose their ability to divide, is accompanied by a specific set of changes including growth cessation, morphological changes, appearance of senescence-associated beta-galactosidase (SA-β-gal) activity and increased expression of cyclin-dependent kinase inhibitors (CDKIs). Though lack of a clear correlation between organismal aging with cellular growth viability, the study of mammalian cell aging in vitro has enormous potential for telling us how human aging works [1]. Besides, it is noteworthy that cellular senescence is well regarded as one of cellular mechanisms to prevent oncogenesis [2].

The silent information regulator 2 (Sir2) is an NAD-dependent deacetylase. It is well known that overexpression of Sir2 or its orthologs can extend organismal life span in a wide range of lower eukaryotes, including yeasts [3], [4], worms [5] and flies [6]. In mammalians, Sir2 is represented by seven homologues (SIRTs 1∼7), of which SIRT1 is the most closely related to the yeast Sir2 and intensively studied. Recent studies have demonstrated that Sirt1 played an important role in the regulation of cell survival by inhibiting apoptosis induced by stresses [7]–[9]. Therefore, it is speculated that SIRT1 can also reduce cell aging. But study of overexpression of seven human sirtuins (SIRT1∼7) failed in demonstrating the effects on replicative life span in skin-derived human cells or prostate epithelial cells [10]. In addition, SIRT1 silencing by RNAi or specific inhibitors did not affect cell viability and was not sufficient to induce activation of endogenous p53 in the absence of applied stress [11]–[13]. However some studies also showed that SIRT1 protein decreased significantly with serial cell passage both in human cells and murine cells and found a significant positive correlation between the level of SIRT1 and cell proliferation and observed an inverse association between SIRT1 and SA-β-gal activity [14]. Besides, there were studies showed that SIRT1 silencing by RNAi could be more sensitive to induce cell arrest in cancer cells than in normal cells [11], [15]. These inconsistencies on the function of SIRT1 in the process of cellular senescence may be associated with cell-type-specific context and different molecular mechanisms involved.

One important mechanism responsible for the replicative senescence of human cells is the erosion and eventual dysfunction of telomeres [16]. However, in certain fibroblasts, e.g. MRC5, WI38 and IMR90, immortalization could not be efficiently obtained only by telomerase transfection [17]. In these cell lines, the accumulation of p16^INK4A^ was noted as another important mechanism that contributes to replicative senescence in these cell lines [18]. Recent studies discovered that the accumulation of p16^INK4A^ may also, in part, contributed to the physiological aging in vivo, for instance the deterioration of age-associated Haematopoietic stem cells (HSC) functions [19], the declines of olfactory bulb neurogenesis [20] and the restraints of islet regenerative potential [21]. Moreover, it was worthy of note that the accumulation of p16^INK4A^ was reported as a robust biomarker in mammals and could be attenuated by caloric restriction (CR) [21]. Therefore it was presumed that the diminished expression of p16^INK4A^ by CR could contribute to the decreased pathology of organs aging. Since SIRT1 acts as a key regulator orchestrating the response to caloric restriction in mammals [22], it enforced us to speculate that the expression of p16^INK4A^ could be partly modulated by SIRT1.

In our study, we aimed at investigating whether increased expression of SIRT1 can regulate cell proliferation and cellular senescence in normal human diploid fibroblasts with feature of p16^INK4A^ accumulation at late passage. Our research demonstrated that SIRT1 could delay cellular senescence and extend cell life span with promoted phosphorylations of ERK and S6K1, which may be partly associated with the expression of p16^INK4A^ in human diploid fibroblasts. Besides, our data revealed that SIRT1/ERK/S6K1 signaling was suppressed in senescent cells, implying the important role in the process of cellular senescence.

## Results

### SIRT1 overexpression led to a reduction of senescence-associated biomarkers

**Figure 1:**
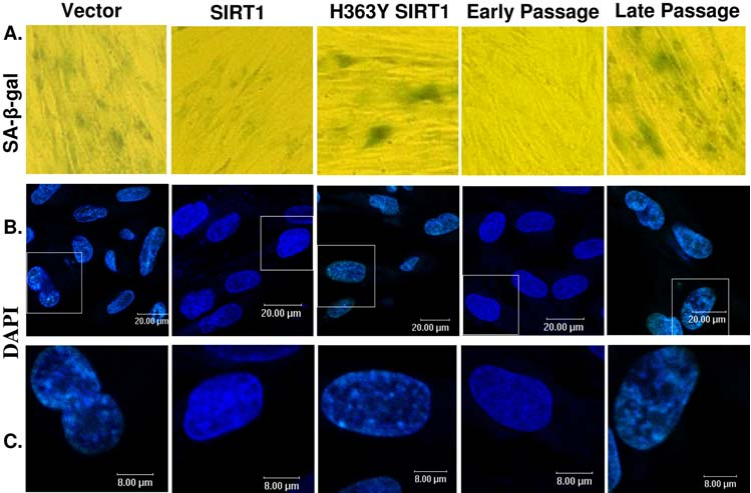
Changes of senescence-associated features in 2BS cells were induced by enforced expression of SIRT1. (A) Stable transfected 2BS cells expressing empty Vector, SIRT1 and H363YSIRT1 were passaged until one of them underwent senescence, then stained for SA-β-gal staining, a classical biomarker for senescence. Early passage (23 PDs) 2BS cells were showed as negative control. Late passage (60 PDs) 2BS cells were stained as positive control. (B) Senescence-associated heterochromatic foci (SAHF), another classical marker of senescence, was showed in 2BS cell lines DNA foci accumulate was visualized by DAPI staining in 2BS cells. Scale bars were equal to 20 µm. (C) Enlarged images of DAPI staining are shown in the lower panels. Scale bars were equal to 8 µm.

Cellular senescence is characterized by elevated level of an endogenous β-galactosidase activity at pH 6.0. To confirm our hypothesis that SIRT1 is associated with cellular senescence, vectors expressing wild type SIRT1 and dominant negative form of SIRT1 (H363YSIRT1), as well as mock vector (pcDNA 3.1) were separately transfected into 2BS, a strain of human embryonic lung fibroblast. After a two-week G418 selection to eliminate untransfected cells, cell populations underwent serial confluent culture with 50 µg/ml G418 until one of them reached notable growth arrest. Subsequently, SA-β-gal activity was measured among different stable transformation pools. Early passage (23 PDs) and late passage (60 PDs) of 2BS cells were also analyzed as controls. **Figure 1A** showed that 2BS with enforced expression of wild type SIRT1 displayed lower frequency of SA-ß-gal staining than cells transfected with mock vector and H363YSIRT1 constructs. But few significant differences in SA-ß-gal activity were observed between mock vector-transfected cells and H363YSIRT1-transfected cells.

The accumulation of Senescence-associated heterochromatin foci (SAHF) is another specific biomarker of senescent cells [16]. As shown in **Figure 1B and C**, senescence induced by extensive passage culture typically displayed punctuated DNA foci which were visualized by DAPI staining. In contrast, exponentially growing human fibroblasts usually displayed several small nucleoli and a more uniform DAPI staining pattern. We observed that 2BS cells expressing SIRT1 did not develop pronounced nucleoli or DNA foci. However mock-transfected and H363YSIRT1-transfected cells appeared prominent DNA foci which are nearly indistinguishable from those in senescent 2BS cells. The appearance of DNA foci in different transformations coincided precisely with SA-ß-gal activity and the onset of senescence.

Consist with the SIRT1-induced reduction of senescence-associated biomarker, SIRT1-tansfected 2BS displayed an increased lifespan while H363YSIRT1-transfected 2BS showed a little decreased life span, as compared with mock-transfected cells and untransfected cells, shown in **Table 1**.

**Table 1:** Cumulative population doublings of SIRT1, H363YSIRT1, Mock-transfected cells and untransfected cells

Cells	Cumulative population doublings(PDs)	Number of test samples
untransfected cells	54–59	6
Mock vector	51–55	5
SIRT1	63–70	6
H363SIRT1	46–53	5

### SIRT1 promoted cell proliferation in 2BS cells

**Figure 2:**
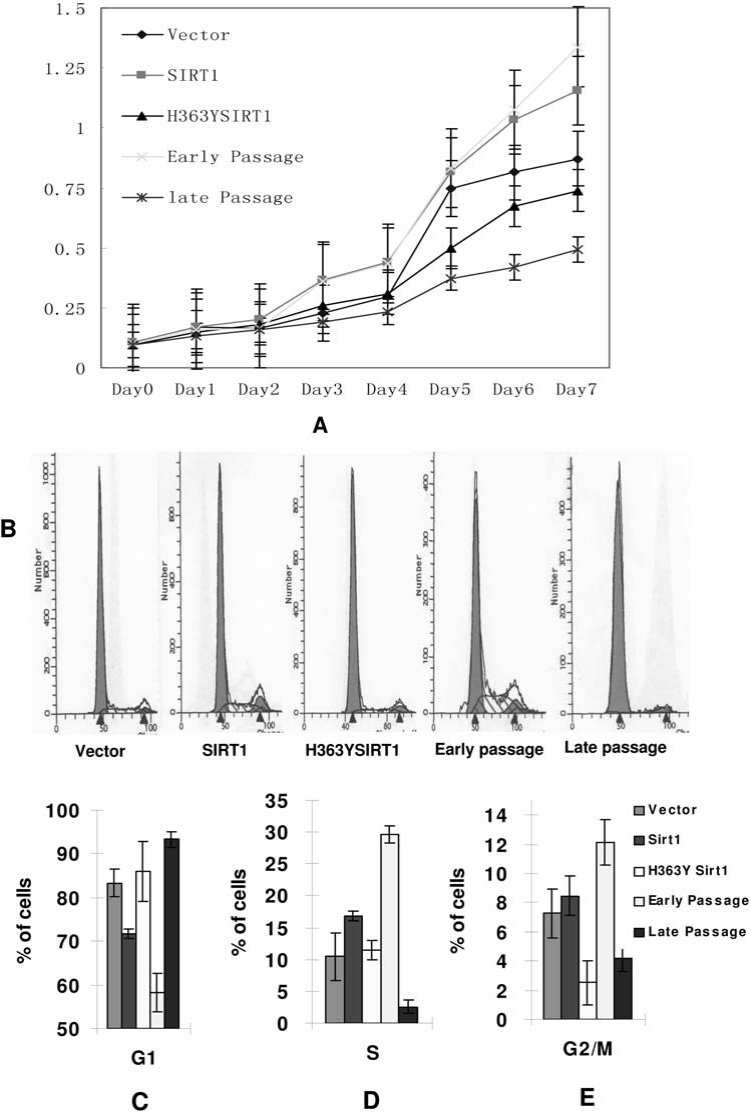
Induced expression of SIRT1 promoted cell proliferation in 2BS cells. (A) Growth curves of transfected 2BS cells (all at PD50) with expression of Vector, SIRT1, and H353YSIRT1. Early passage (23PDs) and late passage cells (58PDs) of 2BS were shown for comparison. Cell proliferation was determined by the MTT assay. Each experiment was performed on 6 independent wells at each time indicated. (B) Flow cytometric analysis of 2BS cells with expression of empty vector, SIRT1 and H363YSIRT1. Early passage (23 PDs) and late passage 2BS cells (58 PDs) were also shown for comparison. Experiments were repeated 3∼4 times with similar results. (C, D, E) Typical cell cycle histograms representing percentage of cells at G1, S, G2/M phases were recorded from 3 replicate experiments. The mean percentage of cells at different phases was showed below.

As cells undergo senescence, their proliferation also decline significantly. To observe the variations in proliferation, the growth rates of different transformations and the control cells were measured by MTT method (**Figure 2A**). The growth curves of 2BS cells expressing SIRT1 advanced quickly even at 50 PDs, displaying a strong proliferation potential similar to young cells (23 PDs), whereas the curves of cells containing mock vector and H363YSIRT1 constructs were approaching to those of senescent cells (58 PDs).

To clarify the mechanisms of SIRT1-drived cell proliferation described above, the cell cycle profiles of transfected and untransfected 2BS cells were analyzed by flow cytometry. Each experiment was performed on 6 independent wells and representative data were shown in **Figure 2B, C, D and E**. Consisted with growth profiles observed in **Figure 2A**, 2BS cells transfected with SIRT1 markedly postponed G1 phase arrest while mock-transfected cells and H363YSIRT1-transfected cells reached G1 phase arrest after confluent culture. In addition, SIRT1 seemed to have less effect on S and G2 phase. Thus, we concluded that enforced SIRT1 expression led to cell proliferation by suppressing G1 arrest and initiated the G1/S transition.

### SIRT1 suppressed cellular senescence through p16^INK4A^/Rb pathway

**Figure 3:**
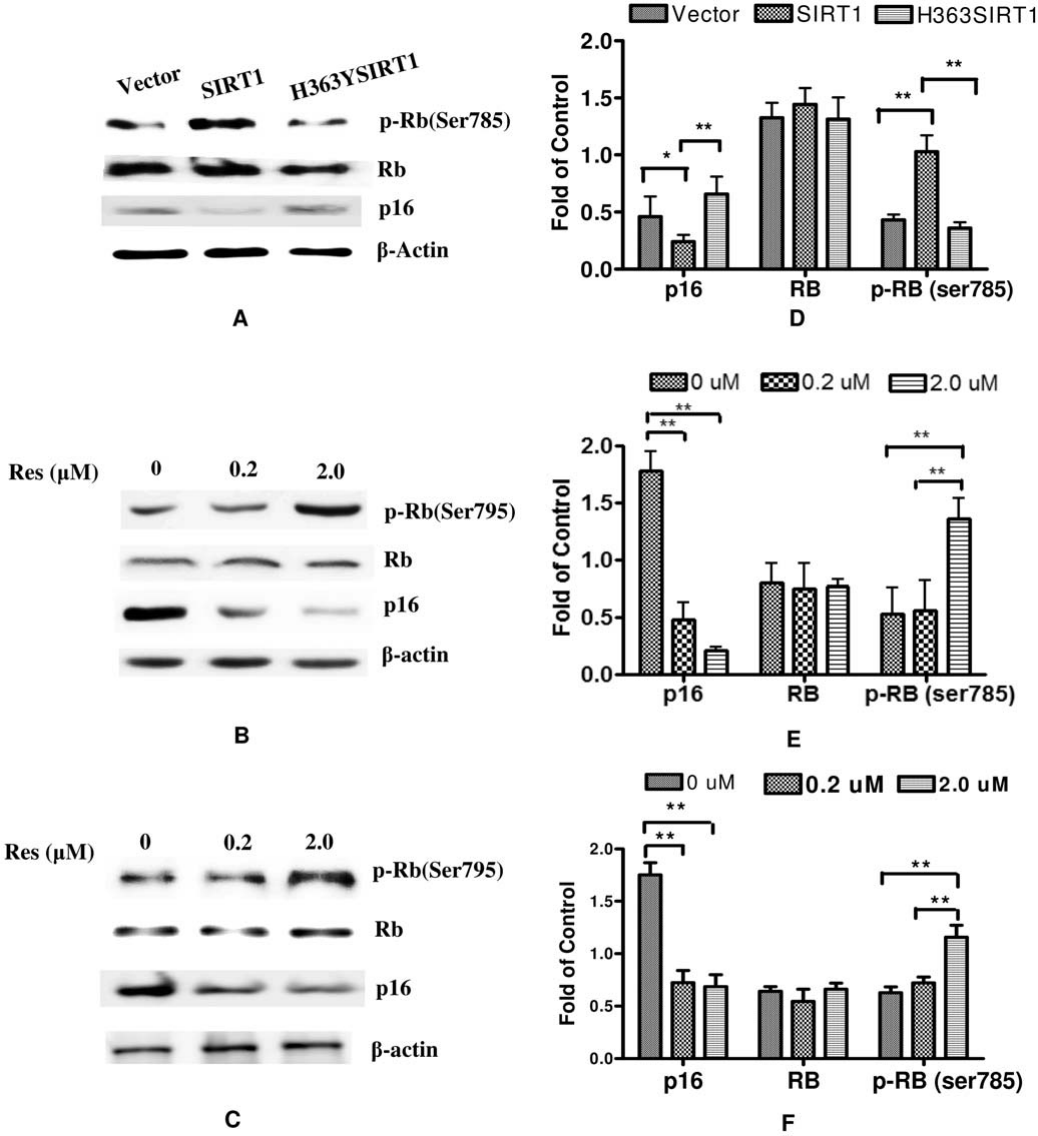
SIRT1-dependend suppression of cellular senescence was through p16^INK4A^/Rb pathway. (A) Transfected 2BS cells containing pcDNA3.1, pcDNA-SIRT1 and pcDNA-H363YSIRT1 were lysated and prepared for western blot analysis by using specific antibodies against phospho-retinoblastoma (Ser795), retinoblastoma, p16^INK4A^ and β-actin. (B, C) 2BS cells and WI38 cells at 40 PDs were treated with solvent alone or 0.2 or 2 µM of resveratrol (Res) for 72 h. Cell lysates were prepared for Western blot with the same antibodies. (D, E, F) The quantitative protein expression was summarized in bar graphs (left) presented by mean values (±SEM) of 3 independent experiments. Error bars represent the S.D. Significant difference compared between controls, *, p<0.05; **, p<0.01.

In 2BS, cellular senescence is largely associated with the inactivation of p16^INK4A^/Rb signaling pathway [23]. Increased p16^INK4A^ prevents activation of the cyclin D/CDK4/CDK6 and subsequently promotes the growth inhibitory functions of RB. As well as the accumulation of p16^INK4A^ in vitro, p16^INK4A^ also displays a robust age-dependent increase in vivo [24]. To explore the mechanisms by which SIRT1 delays cellular senescence in human fibroblasts, p16^INK4A^/Rb signaling pathway was detected subsequently among different transformations. The total cellular proteins were prepared and analyzed by western blot for detecting the expression of p16^INK4A^, Rb, and phosphorylation of Rb at Ser795. **Figure 3A, 3D** indicated that, compared with mock vector and H363YSIRT1 transfected 2BS cells, p16^INK4A^ displayed a two-fold decrease in SIRT1-transfected cells, accordingly phospho-Rb (Ser795) was increased for more than two times whereas the levels of β-actin and Rb remained unchanged in different batches of transfected cells. Resveratrol (Res) is regarded as an activator of SIRT1, promoting the survival of human cells in vitro [25]. To further investigate the role of SIRT1 affecting p16^INK4A^/Rb signaling pathway, resveratrol was used to treat WI38 cells and 2BS cells with different concentrations for 72 hr. As shown in **Figure 3B, 3C**, resveratrol decreased the expression of p16^INK4A^ and increased phosphorylation of Rb (Ser795) in a dose-dependent manner, whereas the levels of β-actin and Rb were invariable in either presence or absence of resveratrol. Accordingly, the quantitative protein expression variations were summarized by bar graph in **Figure 3D, 3E**.

### SIRT1 promoted phosphorylations of kinases associated with cell proliferation

**Figure 4:**
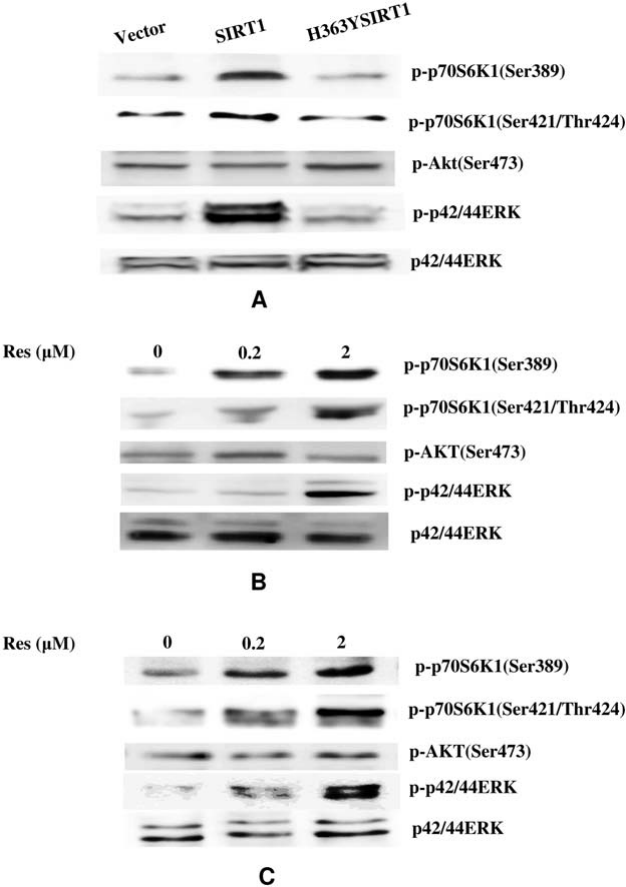
SIRT1-dependend phosphorylation of kinase was associated with the cell proliferation. (A) Transfected 2BS cell containing pcDNA3.1, pcDNA-SIRT1 and pcDNA-H363YSIRT1 were lysated and subjected to western blot analysis by using specific antibodies against Phospho-p44/42 ERK (Thr202/Tyr204), Phospho-AKT (Ser473), Phospho-p70 S6K1(Thr389), Phospho-p70 S6K1 (Thr421/Ser424)and p44/42 ERK. (B, C) 2BS cells and WI38 cells at 40 PDs were treated with solvent alone or 0.2 or 2 µM of resveratrol (Res) for 72 h. Cell lysates analyzed by western blot, as described in Figure 4A.

Our results **(** **Figure 2** **)** showed that SIRT1 promotes cell proliferation in 2BS. And activities of kinase signaling usually contribute to cell viability in human fibroblasts [26]–[29]. To confirm our hypothesis that SIRT1 promotes cell proliferation by activating some kinase signaling, we examined the activation of ERK, AKT and p70 S6K1 in different batches of transformations. As shown in **Figure 4A**, phosphorylations of ERK (Thr202/Tyr204) and S6K1(Thr389, Thr421/Ser424) were enhanced in SIRT1-tansfected 2BS cells, whereas that of AKT (Ser473) was unchanged by SIRT1 overexpression. Compared to mock-transfected cells, 2BS with enforced expression of H363YSIRT1 did not significantly influence the phosphorylations of ERK, AKT and p70 S6K1. **Figure 4A** also showed that total ERK protein levels were not altered in different batches of transformations. To confirm such SIRT1-associated activation of Kinases, resveratrol was used to treat WI38 cells and 2BS cells with different concentrations. As shown in **Figure 4B**, resveratrol increased the phosphorylations of ERK (Thr202/Tyr204) and S6K1(Thr389, Thr421/Ser424)in a dose-dependent manner while that of AKT was not altered in either treated or untreated cells. These data were consistent with the results obtained above from SIRT1-transfected 2BS, suggesting that SIRT1 was associated with the promoted phosphorylations of ERK and S6K1.

### Inhibition of S6K1 activity led to increased p16^INK4A^/Rb pathway

**Figure 5:**
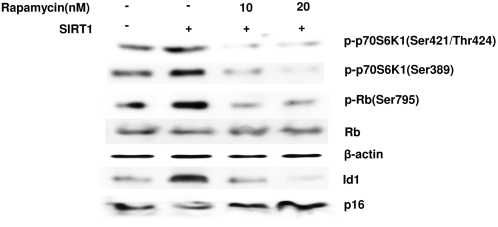
Inhibition of S6K1 activity led to increased p16/Rb pathway with Id1 suppression. SIRT1-transfected 2BS were treated for one day with increasing concentrations of Rapamycin (10nM and 20nM). Following treatment, the cells were lysed and subjected to western analysis for determining phosphorylation of S6k1 and signaling associated with the cell cycle G1 arrest (Id1, p16^INK4A^, Rb and phosphorylated Rb). Transfected 2BS containing SIRT1 and Mock vector were also analyzed as a positive and a negative control. Levels of β-actin were used as a loading control.

Since SIRT1 regulated both p16^INK4A^/Rb pathway and ERK/S6K1 signaling, it will be interesting to investigate whether SIRT1-stimulated S6K1 phosphorylation is associated with the regulation of p16^INK4A^/Rb pathway. SIRT1-transfected 2BS cells were treated with increased concentrations of Rapamycin for inhibiting SIRT1-induced S6K1 phosphorylation. The signaling, e.g. Id1, p16^INK4A^ and Rb, which were observed to lead to 2BS senescence [23], [30], were subsequently detected by western blot. As shown in **Figure 5**, treating SIRT1-transfected cells with Rapamycin resulted in a decreased phosphorylation of S6K1 and Id1 expression, subsequently led to an increase in the expression of p16^INK4A^ and a decreased phosphorylation of Rb at 795 whereas the levels of Rb and β-actin remained unaltered.

### The levels of SIRT1 protein and phosphorylations of ERK and S6K1 were declined in senescent 2BS cells

**Figure 6:**
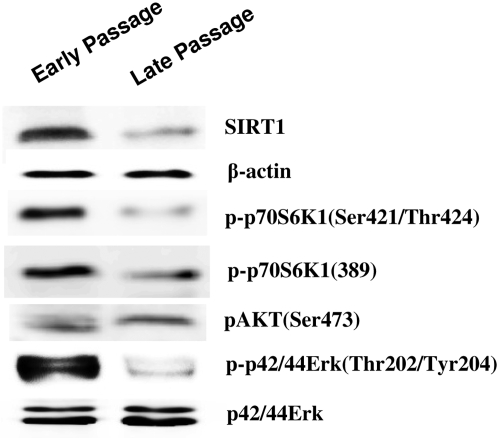
The alterations of SIRT1 expression and phosphorylation of ERK, AKT and S6K1 were determined in senescent 2BS Cells. The total cellular lysates from early passage (23 PDs) and late passage of 2BS (63 PDs) were prepared and subjected to western blot analysis by using specific antibodies against SIRT1,β-actin, Phospho-p44/42 ERK(Thr202/Tyr204), p44/42 ERK, Phospho-p70 S6K1 (Thr389), Phospho-p70 S6K1(Thr421/Ser424)and Phospho-AKT (Ser473).

Having demonstrated that SIRT1-induced ERK/S6K1 signaling was associated with SIRT1-dependent interference of cellular senescence and cell proliferation, it was reasonable to determinate the alterations of SIRT1/ERK/S6k1 signaling in cell aging. As shown in **Figure 6**, the levels of SIRT1 protein were declined in late passage of 2BS (63PDs), and the phosphorylations of ERK and S6K1, but not that of AKT, were also decreased in senescent cells. Our results suggested that the decline of SIRT1/ERK/S6K1 signaling in senescent cells may be for the cell aging in 2BS.

## Discussion

It is well known that overexpression of Sir2 orthologs extends life span in a wide range of lower eukaryotes [3]–[6]. These findings inspired the assumptions that SIRT1 has a role in regulating life span in mammals [29] and as a potential pharmacological target to treat the major diseases of aging [31]. Studies by Michishita et al indicated that overexpression of SIRT1 had no effects on replicative life span of foreskin-derived NHF cells and prostate epithelial cells [10]. But he further documented that SIRT1 was down-regulated in human senescent cells, suggesting that SIRT1 may be required to extend replicative life span. Chua et al. revealed that SIRT1-deficient mouse embryonic fibroblasts (MEFs) had extended replicative life span under sub-lethal oxidative stress, but showed p19 induction and cell cycle arrest upon acute DNA damage [32]. Our results showed that enforced SIRT1 expression in 2BS promoted cell life span and prevented cellular senescence with the features of delayed SA-β-gal staining, reduced SAHF formation and G1 arrest, increased cell growth rate and extended replicate life span. However, H363YSIRT1 might not show any pronounced cellular senescence features but manifested small but significantly decreased life span compared to controls. Studies by Sasak et al [14] documented that the level of SIRT1 was declined significantly with serial cell culture passage in both human and murine cells, and showed a positive correlation with cell proliferation and a negative correlation with SA-β-gal staining. In similar ways, a high SA-β-gal staining was observed in WI38, IMR90 and human endothelial cells induced by sirtinol or splitomicin, SIRT1 inhibitors, by Ota H et al [15], [33]. The inconsistencies on the function of SIRT1 in the process of cellular senescence may depend on cell-type-specific context and different molecular mechanisms involved.

Ota H et al observed that WI38 and IMR90 could be induced into senescence by SIRT1 inhibitor [15]. But detail molecular mechanisms were unknown. Like WI38 and IMR90, the senescence of 2BS was largely associated with the accumulation of p16^INK4A^ at late passage [23], [30]. And anti-sense p16^INK4A^ expression could largely delay the senescence of 2BS [23]. It will enforce us to assume that the SIRT1 activation may prevent 2BS senescence. Furthermore it was documented that age-associated p16^INK4^ accumulation, in some rodent tissues, could be attenuated by CR and SIRT1 acts as a key regulator orchestrating the response to CR in mammals, it promoted the assumption that SIRT1 could partly regulate the expression of p16^INK4A^. These assumptions were consequently confirmed by SIRT1 overexpresion in 2BS and treating 2BS and WI38 cells with dose-dependent resveratrol, a SIRT1 activator. A latest research indicated that p16^INK4A^/Rb pathway was required for SAHF formation and cellular senescence [34]. Consistent with their conclusions, our results indicated that the SIRT1-associated prevention of SAHF formation may be dependent on suppressing p16^INK4A^/Rb pathway. Although there is no data previously reported that SIRT1 could suppressed the expression of p16^INK4A^, there were studies documented that histone deacetylase inhibitors (HDACIs) sodium dibutyrate (SDB) and trichostatin A (TSA) induced senescence in human cells by regulating p16^INK4A^/Rb pathway [35], indicating that SIRT1 may suppressed the expression of p16^INK4A^ in a similar way. Since the p16^INK4A^ expression was reported as a robust in vivo biomarker in human and rodent with advancing age [21], [24], the suppression of p16^INK4A^ by SIRT1 may benefit to attenuate age-related diseases.

Activation of ERK, AKT and p70 S6K is required for cell proliferation. The inhibition of such kinases could induce cell arrest in fibroblasts [26]–[28]. Our data showed that both SIRT1 transfection and Resveratrol treatment promoted phosphorylations of ERK (Thr202/Tyr204) and S6K1 (Thr389, Thr421/Ser424), whereas that of AKT (Ser473) was unchanged. Consistent with our results, SIRT1 was reported to induce the phosphorylation of ERK in HEK293 cells [36]. Though no works illuminated the mechanisms of SIRT1-induced phoshorylation of ERK, Kobayashi et al documented that HDAC inhibitor FK228 suppressed the MAP kinase signaling pathway by upregulating Rap1 transcription [37], suggesting that SIRT1 might recruit a similar way to provoke ERK signaling.

S6k1 was required for cell growth and G1 cell cycle progression and involved in translational control of 5′ oligopyrimidine tract mRNAs via promoting the phosphorylation of S6 protein of the 40S ribosomal subunit [38]. These indicated that SIRT1 may be functionally for the mRNA translation by activating S6K1 phosphorylation. Recent works issued that the tumor suppressor protein tuberous sclerosis complex 2 (TSC 2), an upstream regulator of mTOR, could be phosphorylated by ERK signaling [39], [40], indicating that the phosphorylation of S6K1 may be mediated by ERK signaling.

Since the activation of S6K1 was obligatory for cell cycle progression and the inhibition of such enzyme led to cell cycle arrest [41], [42], it was presumed that S6K1 may be associated with SIRT1-dependent p16^INK4A^ decline in 2BS. Latest data revealed that mTOR, a direct upstream signaling for the phosphorylation of S6k1, was for the induction of Id1 expression in mammary epithelial cells. Moreover Id1 was responsible for the down regulation of p16^INK4A^ in human diploid fibroblasts [30], [43]. These researches suggested that there will be a potential association between S6K1 and p16^INK4A^. Our data showed that, after treating SIRT1-transfected 2BS with dose-dependent Rapamycin, phosphorylation of S6K1 and Id1 expression was suppressed, subsequently p16^INK4A^/Rb pathway was provoked. These results implied that SIRT1-induced phosphorylation of S6K1 may be responsible to the accumulation of p16^INK4A^ via the introduction of Id1. But further witnesses should be investigated by S6K1 RNA interfere approach or transfecting dominant negative S6K1 mutaition.

It was worthy of note that SIRT1 expression and the phosphorylations of ERK and S6K1 were decreased in senescent 2BS. The decline of SIRT1/ERK/S6K1 in the late of passage may be responsible for the senescence of 2BS. The elimination of S6K1 phosphorylation in senescent fibroblasts was previously reported by Zhang et al [44]. The variations of ERK signaling in senescent cells still remained disruptions [43], [45]. The inconsistencies may result from the use of different cell lines or different culture conditions used.

**Figure 7:**
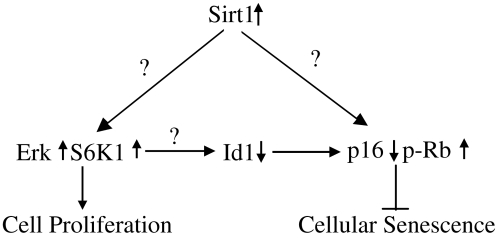
Schematic model for a mechanism of Sirt1 activating ERK/S6K1 signaling pathway involved in cell senescence. The activation of SIRT1 in human lung fibroblasts suppressed p16^INK4A^/Rb pathway and provoked ERK/S6K1 signaling, which, in turn, may inhibit p16^INK4A^/Rb signaling via Id1 induction.

*In summary*, our data suggested that, in the presence of stress, overexpression of SIRT1 contributed to cell proliferation and prevention of cellular senescence in human diploid fibroblasts. The delay of senescence by SIRT1 was associated with down-regulation of p16^INK4A^/Rb pathway and the activation of ERK/S6K1 signaling. The decline of SIRT1-dependent ERK/S6K1 signaling in senescent cell may be contribute to cell progression loss and cellular senescence at late passage of human diploid fibroblasts. Based on above evidence we have put forward a possible model of how SIRT1 antagonize cellular senescence via promoted ERK/S6K1 singling, shown in **Figure 7**.

## Materials and Methods

### Cell Culture and Synchronization

2BS cell line was isolated from female fetal lung fibroblast tissue in the National Institute of Biological Products, Beijing, China. The characters of 2BS have been fully characterized since its isolation in 1962 [26]–[28]. The maximal life span of 2BS is about 60 population doublings (PDs). 2BS cells are considered to be young below 30 PDs and go into senescence at 55 PDs or above. Cells were maintained in Dulbecco's modified Eagle's medium (Invitrogen) supplemented with 10% fetal bovine serum, 100 units/ml of penicillin and 100 µg/ml streptomycin at 37°C in 5% CO2. WI38 cell line was cultured in the same way.

For synchronization, human fibroblasts were rendered quiescent by serum deprivation for 48 hr and then stimulated to reenter the cell cycle by the addition of serum to a final concentration of 10%. G1 phase cells were harvested at 8 hr after serum stimulation.

### Antibodies and Reagents

Antibodies against p44/42 ERK (#9102), Phosphorylated p44/42 ERK (Thr202/Tyr204) (#9101), Phosphorylated Rb (Ser795) (#9301), Rb (4H1) mouse Antibody (#9309), Phosphorylated S6K1 (Thr389, Thr421/Ser424) (#9430) were purchased from Cell Signaling Technology (Beverly, MA). p-Akt123 (Ser473) Antibody (SC7985-R), SIRT1 (H300) Antibody (SC15404), β-actin (I-19) Antibody (sc-1616) and Id1 (C-20) Antibody (sc-488) were purchased from Santa Cruz Biotech., Inc. p16^INK4A^ (Ab-1) Antibody (#MS-218-P1) was purchased from NeoMarkers Corp. Resveratrol (R5010) was purchased from Sigma-Aldrich Cop. Rapamycin (#553210) was from Calbiochem. Other chemicals and reagents were of the purest grade available.

### Stable Transfection of Human Diploid Fibroblasts

pcDNA SIRT1, pcDNA H363YSIRT1 and pcDNA 3.1B were transfected into young 2BS cells at 20 PDs with Lipofectamine 2000, performed according to standard method. After 48 hr, the cells were selected by G418 (100 µg/ml; Life Technologies). Colonies of stable transformations were isolated 3∼4 weeks later and propagated accordingly in complete medium containing 50 µg/mL G418. The resulting transformations were termed as SIRT1, H363YSIRT1 and Vector respectively.

### Senescence-associated beta-Galactosidase Staining

Transfected 2BS and untransfected early passage and late passage 2BS cells were washed twice in PBS, fixed to plates using 3% formaldehyde for 3∼5 min, and washed with PBS again. Then these cells were incubated overnight at 37°C without CO2 in a freshly prepared staining buffer (1 mg/ml X-gal, 40 mM citric acid/sodium phosphate, pH 6.0, 5 mM potassium ferrocyanide, 5 mM potassium ferricyanide, 150 mM NaCl, 2 mM MgCl2) [40].

### Senescence-associated beta-heterochromatic foci (SAFH) analysis

To determine SAFH formation, cells were cultured directly on glass cover slips and then fixed with 4% Para formaldehyde. After washing with PBS, Cells were permeabilized with 0.2% Triton X-100/PBS for 10 min. DNA was visualized by DAPI (1 µg/ml) for 1 min, and then washed with PBS for twice. Cover slips were mounted in a 90% glycerol PBS solution. Cover slips were examined under Leica DMIRB inverted microscope.

### MTT Growth Curve

2BS cells were detached and seeded into 96-well plates 2500 cells per well. At the indicated times, cells were stained with 3-(4,5-dimethylthiazol-2-yl)-2,5-diphenyl tetrazolium bromide (MTT) (10 µg/ml) in PBS for 3 hr, then dissolved with 50% *N*,*N* dimethylformamide and 10% SDS for 3hr at 37°C. The optical density at 570 nm was determined. Each point was determined in sextuplet.

### Cell Cycle Analysis

When cells reached 70–80% confluence, they were washed with PBS, detached with 0.25% trypsin, and fixed with 75% ethanol overnight. Samples were resuspended in 0.5 ml PBS and stained with propidium iodide in the dark for 30 min, and the DNA contents were measured by fluorescence-activated cell sorting (FACS) on a Becton-Dickinson FACScan flow cytometry system. The data were analyzed using a CellFIT software.

### Examination of Replicative Lifespan

All transfected 2BS cells and untransfected 2BS cells were subcultured at a split ratio of 1:2. The number of cells was counted at each passage, and the number of population doublings (PDs) achieved between passages was determined by log_2_ (number of cells obtained/number of cells inoculated). When the cells stopped dividing and remained impossible to be further passaged for 2 weeks, they were regarded as growth-arrested. Conclusions were confirmed by two independent transduction tests

### Western Blot

Cells were lysed in radioimmune precipitation assay buffer (1×PBS, 1% Nonidet P-40, 0.5% sodium deoxycholate, 0.1% SDS, 100 µg/ml phenylmethylsulfonyl fluoride, 50 kallikrein-inactivating units/ml aprotinin, and 1 mM sodium orthovanadate). Protein concentration of each sample was determined by BCA Protein Assay Reagent (Pierce). 100 µg of proteins was loaded on 15% SDS-polyacrylamide gel and transferred to polyvinylidene difluoride filters (Millipore). The filter was blocked and then incubated with the primary antibody in 5% nonfat dry milk in TBST (10 mM Tris-Cl, pH 7.5, 150 mM NaCl, 0.05% Tween 20) overnight at 4°C. After washing, the blots were incubated with secondary antibody (1 µg/ml) conjugated to horseradish peroxidase at 1:2,500 in TBST for 1 hr at room temperature. Proteins were visualized with Chemiluminescent Substrate (Pierce) according to the manufacturer's instruction.
